# Bioactive Metabolites from Terrestrial and Marine Actinomycetes

**DOI:** 10.3390/molecules28155915

**Published:** 2023-08-06

**Authors:** Chananan Ngamcharungchit, Nutsuda Chaimusik, Watanalai Panbangred, Jirayut Euanorasetr, Bungonsiri Intra

**Affiliations:** 1 Department of Biotechnology, Faculty of Science, Mahidol University, Bangkok 10400, Thailand; 2 Mahidol University and Osaka University Collaborative Research Center on Bioscience and Biotechnology, Bangkok 10400, Thailand; 3 Research, Innovation and Partnerships Office, King Mongkut’s University of Technology Thonburi, Bangkok 10140, Thailand; 4 Department of Microbiology, Faculty of Science, King Mongkut’s University of Technology Thonburi, Bangkok 10140, Thailand; 5 Laboratory of Biotechnological Research for Energy and Bioactive Compounds, Department of Microbiology, Faculty of Science, King Mongkut’s University of Technology Thonburi, Khet Thung Khru, Bangkok 10140, Thailand

**Keywords:** actinomycetes, bioactive metabolites, biodiversity, genome mining, silent gene activation

## Abstract

Actinomycetes inhabit both terrestrial and marine ecosystems and are highly proficient in producing a wide range of natural products with diverse biological functions, including antitumor, immunosuppressive, antimicrobial, and antiviral activities. In this review, we delve into the life cycle, ecology, taxonomy, and classification of actinomycetes, as well as their varied bioactive metabolites recently discovered between 2015 and 2023. Additionally, we explore promising strategies to unveil and investigate new bioactive metabolites, encompassing genome mining, activation of silent genes through signal molecules, and co-cultivation approaches. By presenting this comprehensive and up-to-date review, we hope to offer a potential solution to uncover novel bioactive compounds with essential activities.

## 1. Introduction

Actinomycetes are generally recognised as filamentous Gram-positive bacteria in the order *Actinomycetales* [1]. Typical actinomycete colonies show distinct powdery characteristics containing filamentous mycelium-like fungi and spore-forming properties (e.g., genera *Streptomyces*, *Microbispora*, *Streptosporangium*, and *Microbispora*). The genus *Streptomyces* stands out as the most dominant and well-known actinomycetes. These are primarily aerobic bacteria with a notable high G + C content in their DNA, approximately ranging from 60% to 78% [1,2]. The *Actinomycetales* members show extensive diversity in their morphology, physiology, and metabolic capabilities. Rare or non-streptomycete actinomycetes are a group of actinomycete bacteria that are rarely isolated from the environment compared to dominant *Streptomyces*. Rare actinomycetes include *Actinomadura*, *Actinoplanes*, *Actinokineospora*, *Actinosynema*, *Kineosporia*, *Planobispora*, *Nocardia*, *Thermomonospora*, *Saccharothrix*, and *Saccharopolyspora* [3]. Rare actinomycetes have attracted attention due to their low frequency in isolation and the potential discovery of new natural bioactive compounds such as macrolide antibiotics [4,5].

**Figure 1:**
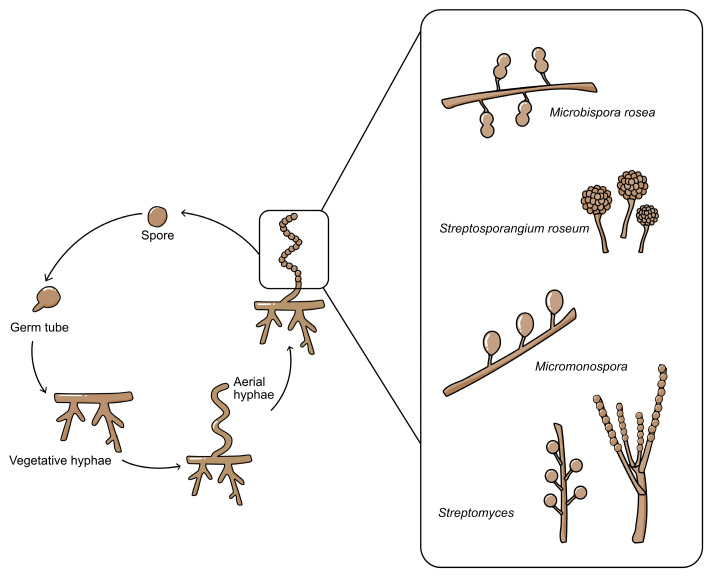
The life cycle of actinomycetes, starting from conidiospores until sporulation. The various types of conidiospores are shown in the expanded box.

Most actinomycetes are aerobic, saprophytic microorganisms with complex life cycles (Figure 1) except for unicellular *Corynebacterium* and *Mycobacterium* [6]. Actinomycetes have well-developed radial mycelium dividing into the substrate and aerial mycelium during their life cycle. Substrate mycelium is developed in the media to assimilate nutrients, and then aerial mycelium is developed afterwards. However, when actinomycetes grow in an impoverished environment, the hypha becomes coiled and develops a septum. After the septum develops, conidiospores form within the hyphae. Except for *Streptomyces* with its long-chain spores, other genera have distinguishable characteristics, such as *Micromonospora* (single non-motile spore), *Microbispora* (two spores in a chain), and *Streptosporagium* (bearing sporangium as spore vesicle) [1]. Spores will be released into the environment and germinate when favourable condition is achieved. In the germination step, the spore will protrude germ tubes then, germ tubes reach the vegetative growth stage, and the cycle is repeated [2,7].

Actinomycetes play an important role in biotechnology and pharmacology because they can produce various secondary metabolites and other useful compounds such as antibiotics, antitumor agents, immunosuppressive agents, and nutritional materials. Therefore, their secondary metabolites and diversity make actinomycetes an important group of key compound producers. This review focuses on the recent bioactive metabolites derived from terrestrial and marine actinomycetes from the years 2016 to 2023. Additionally, the methodology for discovering new metabolites is also discussed.

## 2. Ecology of Actinomycetes

**Figure 2:**
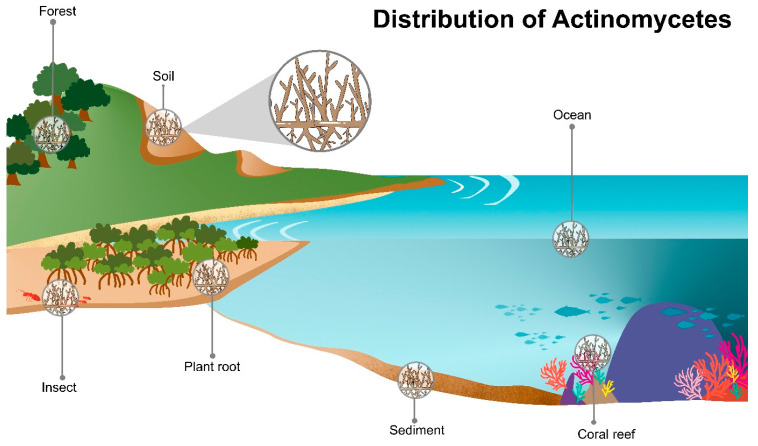
Diversity of actinomycetes habitats. Actinomycetes are predominantly found in various ecological habitats e.g., marine ecosystems (of water bodies, coral reefs, seawater, mangrove forest), and terrestrial ecosystems (soil and plants, and insects).

Actinomycetes are widely distributed in various ecosystems and habitats including soil and marine habitats, freshwater, animals, plants, insects, and fertilizer (Figure 2) [8,9]. They are free-living, saprophytes in an environment such as soil pore [8] or living as endophytes in plants [10]. The actinomycete members include inhabitants of soil or aquatic environments (e.g., *Streptomyces*, *Micromonospora*, *Rhodococcus*, and *Salinispora* species); plant symbionts (e.g., *Frankia* spp.); and insect, plant, or animal pathogens (e.g., *Corynebacterium*, *Mycobacterium* or *Nocardia* species) [1,11,12]. They also present in extreme environments especially the psychrophilic area, for example, Antarctica terra and desert soil [13,14,15].

### 2.1. Soil Actinomycetes

Actinomycetes grow as hyphae-like fungi responsible for the characteristically “earthy” smell of freshly turned healthy soil. The actinomycete population is largest in the surface layer of soils and gradually decreases with depth because of their need for oxygen [16,17]. Their estimated values range from 10^4^ to 10^8^ cells per gram of soil. They are sensitive to acidity/low pH (optimum pH range is 6.5–8.0) and waterlogged soil conditions. These microorganisms are primarily mesophilic, thriving in temperatures between 25 °C and 30 °C. Actinomycetes play a crucial ecological role as saprophytes, actively participating in various biological processes such as organic matter recycling, bioremediation, and promoting plant growth.

Plant growth-promoting actinomycetes use both direct (e.g., producing plant hormones) and indirect (e.g., inhibitory to plant pathogens) mechanisms to influence plant growth and protection [18,19,20,21]. They impact the process of plant biomass decomposition [22] and the microbiome around the rhizosphere [20]. They are also found in salty soil samples [23]. Ahmed et al. [24] explored the saline soil microbiome for its native structure and novel genetic elements involved in osmoadaptation. Their 16S rRNA gene sequence analysis indicated the dominance of halophilic/halotolerant phylotypes affiliated with *Pseudomonadota*, *Actinomycetota*, *Gemmatimonadota*, *Bacteroidota*, *Bacillota*, and *Acidobacteriota*. Their microbiome analysis revealed that the abundance of *Actinomycetota* among the other phyla was 21%.

### 2.2. Endophytic Actinomycetes

Endophytic actinomycetes that reside within the interior tissues of healthy plants without adversely affecting the host plant are an excellent source of potential new bioactive compounds [10,12]. Endophytic strains have been isolated from diverse plants, such as crop plants, medicinal plants, halophytes, and some woody tree species [10,25]. Examples of endophytic actinomycetes include *Actinoallomurus*, *Actinoplanes*, *Allonocardiopsis*, *Amycolatopsis*, *Blastococcus*, *Glycomyces*, *Kibdelosporangium*, *Micrococcus*, *Micromonospora*, *Modestobacter*, *Nocardia*, *Nocardioides*, *Nonomuraea*, *Plantactinospora*, *Pseudonocardia*, *Pseudonocardia*, *Rothia*, *Saccharopolyspora*, *Solirubrobacter*, *Sphaerisporangium*, *Streptomyces*, *Streptosporangium*, *Wangella*, and *Xiangella* [12].

A 16S rRNA analysis by Janso and Carter [26] categorised 113 isolated actinomycetes from 256 tissue samples (e.g., leaves, roots, and stems) collected from 113 plants. They found six families: *Streptosporangiaceae* (40%), *Streptomycetaceae* (27%), *Thermomonosporaceae* (16%), *Micromonosporaceae* (8%), *Pseudonocardiaceae* (8%), and *Actinosynnemataceae* (2%). Their findings indicate that rare actinomycetes (non-*Streptomyces*) predominate in plant samples. In general, the appearance of isolated, rare actinomycetes from soil samples will be either inhibited or hindered by fast-growing *Streptomyces* strains [27]. However, this is not the case in plant roots where the ratio of *Streptomyces* is low. Therefore, plant roots are an excellent potential source of rare actinomycetes and, probably, new secondary metabolites.

### 2.3. Actinomycetes in Compost

During the composting process, microorganisms (bacteria, actinomycetes, and fungi) are vital for organic matter degradation to produce carbon dioxide, water, heat, and humus and the relatively stable organic product as byproducts [28]. Composting generally proceeds through three main phases: (i) the mesophilic phase, (ii) the thermophilic phase, and, finally, (iii) the cooling and maturation phase. Different microbial consortia play a role during each composting phase [29]. Early decomposition is performed by mesophilic microbes, which rapidly break down soluble, readily degradable compounds. The accumulative heat causes the compost temperature to rise rapidly. As the temperature increases > 40 °C, the mesophilic microorganisms are less active and are replaced by thermophilic microbes. During the thermophilic phase, high temperatures accelerate the degradation of organic molecules such as proteins, fats, and complex carbohydrates in plant-like cellulose, hemicellulose, and lignin [30]. As these complex compounds are used up, the temperature of the compost gradually decreases, and mesophilic microorganisms once again predominate the final phase of ‘curing’ or maturation of the remaining organic matter.

Actinomycetes are typically present in the compost, particularly in the thermophilic and curing stages [31,32]. Thermophilic actinomycetes are well-known components of composts’ microflora and play an important role in habitats where organic matter decomposition occurs at elevated temperatures and under aerobic conditions (e.g., improperly stored hay, cereal grains, manure, straw, and various composts). Thermophilic actinomycetes include various genera, such as *Saccharomonospora*, *Saccharopolyspora*, *Streptomyces*, *Thermoactinomyces*, *Thermobifida*, and *Thermomonospora*.

### 2.4. Marine Actinomycetes

Marine habitats are a rich source of diverse and mostly uncharacterised actinomycetes. Marine habitats include coastal, deep-sea sediment, seawater, and mangrove forests. Mangrove forests are highly dynamic ecosystems that cover 75% of the world’s tropical climate, and the diversity of mangrove organisms remains less unexplored. Mangrove forests are a unique environment because they fluctuate with salinity and tidal gradients that favour their microorganisms to produce unusual metabolites [33].

Reports on marine actinomycetes have emerged since the late 19th century. Since 1980, biotechnology initially provided direction to study marine microorganisms for various applications such as drug development [34]. This study was ongoing until 1984 and used various techniques, identifying the first marine actinomycete, *Rhodococcus marinonascens* [11]. In 2005, the first seawater-obligate marine actinomycetes genus, *Salinispora*, was described. *Salinispora tropica* and *Salinispora arenicola* are novel species belonging to the family *Micromonosporaceae.* This genus required seawater for its growth [35]. Fencial and Jensen [36] detected novel secondary metabolites produced by *Salinispora*, leading to a further search for new groups of marine actinomycetes [34]. Studied marine actinomycete genera include *Dietzia*, *Rhodococcus*, *Streptomyces*, *Salinispora*, and *Micromonospora*. Microbiomes or traditional bacterial enumeration have been studied in marine ecosystems, including seawater [37], coral reefs [38,39,40], and mangroves [36,41,42,43]. There are various groups of microorganisms in mangrove sediments, such as Pseudomonadota, Actinomycetota, and fungi. Both *Streptomyces* and *Micromonospora* from marine habitats are good candidates for isolating potent growth-inhibiting compounds and antitumor agents. Its secondary metabolites show diverse bioactivities, such as antifungal, antitumor, and antibacterial [44]. Marine actinomycetes are one potential marine organism that can produce efficient anticancer agents such as salinosporamide A (*S. tropica*), actinomycin D (*Streptomyces parvulus*), mitomycin C (*Streptomyces caespitosus*), and Rakicidin D (*Streptomyces* sp. MWW064) [45,46].

## 3. Taxonomy and Classification

Early taxonomic systems for actinomycetes relied primarily on phenotypic traits, using similarities and differences in colour (spores, substrate mycelium, and soluble pigments) and melanin pigment production [47,48]. While morphological characteristics are important for describing taxa, they cannot differentiate between many genera. Polyphasic taxonomy was introduced to provide stable nomenclature and reliable identification [49]. The polyphasic approaches are based on integrating genotypic, phenotypic, and chemotaxonomic data obtained using several techniques. Currently, genotypic methods examine nucleic acids (DNA or RNA), while chemotaxonomic methods examine proteins, phenotypic features, and observable characteristics. Each method has a specific taxonomic resolution, offering different discriminatory powers at a particular level in the hierarchy. While a broad range of techniques can be readily performed, not all must necessarily be harnessed. For example, some methods for studying DNA, such as DNA base composition analysis and DNA–DNA hybridisation (DHH), are widely used to characterise bacteria. However, information from other arduous, time-consuming, technologically challenging methods, such as amino acid sequencing, is practical and only needed for a restricted number of taxa [50].

An updated actinomycete taxonomy is based on 16S rRNA- and whole genome-based phylogenetic trees. DDH values have been used for determining relatedness between closely related bacterial species. It is an available technique that offers a genuinely genome-wide distance between organisms. Wayne et al. [51] proposed a DDH value of 70% as the recommended threshold for related species, with a value of ≤70% indicating that the assayed organism belongs to a different species from the reference strains. Genomic similarity can be interpreted from the melting temperature by calculating a digital DDH (dDDH). This genome-based method was expanded by establishing several approaches in addition to BLAST to analyse high-scoring segment pairs, which are used to calculate the average nucleotide identity (ANI) and the conserved percentage of DNA. This technique correlates better with 16S rRNA gene sequences than DDH values and has a lower error ratio. The boundaries of ANI and dDDH values acceptable to propose new species are 95~96% and 70%, respectively [52].

Furthermore, molecular taxonomy, microscopic morphology and chemotaxonomy are essential characteristics to investigate the taxonomy of actinomycetes at the family and genus levels. These characteristics generally relate to the structure of the cell wall and the whole-cell sugar distribution. However, phospholipid composition and menaquinone type may also be considered for fine-tuning purposes [53]. Actinomycetes have diverse morphologies, differing mainly in the presence or absence of a substrate mycelium or aerial mycelium, the colour of the mycelium, the production of diffusible melanoid pigments, and the structure and appearance of the spores [1]. These characteristics are useful for their classification. From the above combination approaches, our group has discovered many new species of rare actinomycetes from diverse habitats, such as *Actinokineospora bankokensis* [54], *Saccharopolyspora rhizosphaerae* [55], *Streptosporangium jomthongense* [56], *Saccharomonospora colocasiae* [57] from rhizosphere soil, *Amycolatopsis iheyensis* [48], and *Micromonospora pelagivivens* [58] from marine sediments.

## 4. A Crucial Secondary Metabolite Producer

Actinomycetes are estimated to be the primary producer of antibiotics among all microbes and produce ~55% of all established antibiotics. Approximately 75% of these were discovered from *Streptomyces*, and the remaining 25% from non-*Streptomyces* species. Of the 22,000 known microbial secondary metabolites, 70% are produced by actinomycetes, 20% by fungi, 7% by *Bacillus* spp., and 1–2% by other bacteria [59].

Antibiotic production by streptomycetes is usually accumulated in a growth-phase-dependent manner. Typically, antibiotics from streptomycetes are produced in small amounts at the transition phase of conidial development when vegetative mycelium growth is slowing due to nutrient exhaustion and aerial mycelium development at the expense of nutrients released by breaking down the vegetative hyphae [60]. Typically, actinomycete secondary metabolites are structurally biosynthesised by polyketide synthase (PKS) and/or non-ribosomal peptide synthetase (NRPS). The genes governing the biosynthesis of secondary metabolites are generally clustered, with secondary metabolites synthesised from their precursors through multiple-step biosynthetic pathways [61].

The secondary metabolites of marine actinomycetes can be classified based on their chemical structure as alkaloids, peptides, polyketides, caprolactones, butanolides, polycyclic xanthones, trioxacarcins, and others. Below, we attempt to summarise the most recent findings for each bioactivity from actinomycetes from between 2016 to 2022, from the PubMed and Web of Science databases.

## 5. Biological Activity of Secondary Metabolites from Actinomycetes and Their Applications

### 5.1. Antibacterial Agents

According to the World Health Organization, the emergence of multidrug resistance by various bacterial pathogens is currently one of the biggest threats to global health and food security [62]. Therefore, finding new antibiotics remains important for expanding alternative metabolites to fight against these bacterial pathogens. Diverse actinomycete antibiotics are listed according to each class such as β-Lactams (penicillin, cephalosporines, carbapenems, monobactam, and Penicillin Binding Protein (PBP) inhibitors, ansamycines, macrolides, tetracyclines and aminoglycosides and antibiotic peptides [63].

Since 2010, our research group has discovered actinomycin production by the rhizosphere *Streptomyces* sp. SJE177 with activity against multidrug-resistant *Staphylococcus aureus* [64] and successfully purified decatromicin derivatives with anti-*Clostidium* activity from rhizosphere *Actinomadura* sp. 2EPS [65]. Currently, more antibacterial metabolites were further discovered from actinomycetes. Table 1 shows that most antibacterial metabolites were identified from *Streptomyces*. The remaining were found from rare actinomycetes from the genera *Actinoallourus*, *Actinomadura*, *Amycolatopsis*, *Kibdelosporangium*, *Kitasatospora*, *Kocuria*, *Microbacterium*, *Micromonospora*, *Rhodococcus*, *Nocardiopsis*, *Pseudonocardia*, *Streptomonospora*, *Streptosporangium*, *Thermoactinomyces*, and *Verrucosispora*.

### 5.2. Antifungal Agents

Among the different types of drugs on the market, antifungal antibiotics, which have an important role in controlling fungal infections, are relatively small but significant groups of drugs. Antifungal agents are widely applied in humans, medicine, agriculture, and veterinary medicine. There are five major classes of antifungal compounds: (i) polyene antibiotics, (ii) allylamines and thiocarbamates, (iii) azole derivatives, (iv) morpholines, and (v) nucleoside analogues [154]. In the late 1950s, the first polyene macrolide antifungal antibiotic from *Streptomyces* species was discovered [155,156]. Polyene antifungal compounds such as amphotericin B are the standard therapy for fungal infections. The mode of action of amphotericin B is its interaction with membrane sterol, which results in aqueous pore production with the polyene hydroxyl residues facing inward, leading to altered permeability, leakage of vital cytoplasmic components, and the death of the organism [157]. The azole, allylamine, and thiocarbamate classes target ergosterol, the same as polyene antibiotics. The morpholine class inhibits sterol synthesis, and the nucleoside analogue class targets DNA synthesis [154]. Most recent antifungal metabolites were identified from *Streptomyces*. The remaining were found in rare actinomycetes from the genera *Actinomadura*, *Amycolatopsis*, *Actinokineospora*, *Norcardia*, *Pseudonocardia*, *Saccharothrix*, and *Umezawaea* (Table 2). Our research group published the antifungal activities of bafilomycin from the rhizosphere *Streptomyces* sp. SBI034 [158] and thailandins from *Actinokineospora bangkokensis* strain 44EHW^T^ [159] against *Colletotrichum* spp., the causative agent of anthracnose disease. Another study showed that adding a halogenated benzoyl group to the benzoate derivative’s 3-OH group improved altholactone’s antifungal activity against *Cryptococcus neoformans* and *Saccharomyces cerevisiae* [160].

### 5.3. Immunosuppressive Agent

Immunosuppressants are essential drugs that significantly decrease the risks of rejecting a transplanted organ. In addition, immunosuppressant drugs are also used to treat many autoimmune disorders, such as Crohn’s disease (chronic inflammation of the digestive tract), rheumatoid arthritis, and patchy hair loss (alopecia areata). Since 1970, the peptide antibiotic cyclosporine A has been the primary immunosuppressant used in transplantation. Various immunosuppressant drugs originally isolated as antifungal antibiotics are produced by microorganisms, such as ascomycin from *Streptomyces hygroscopicus* and tacrolimus from *Streptomyces tsukubaensis* [186]. Regarding their mode of action, immunosuppressant drugs act by binding to immunophilin, which is involved in T-cell activation and proliferation [187].

### 5.4. Biocontrol Agents

Global attempts to discover natural products as biocontrol agents for plant protection have notably increased. Being the most proactive, *Streptomyces* appear to be a readily available natural choice for finding new ways to combat plant pathogens and show appreciable biocontrol action against diverse phytopathogens. However, only a few have been developed as commercial products for plant applications in agriculture. Table 3 lists the microbial pesticides registered in countries worldwide.

Actinomycetes are known for improving compost quality and increasing its nutrient content. They also increase the odour of compost since they can completely digest the organic matter present in compost [188]. It has been shown that the thermophilic Actinomycetota *Streptomyces* sp. No. 101 and *Micromonospora* sp. No. 604 can completely degrade yeast debris and sanitise the compost [189,190]. In addition, *Streptomyces thermodiastaticus* was found to produce various extracellular enzymes involved in pathogenic yeast cell lysis, such as *Candida albicans*. Some thermophilic Actinomycetota can suppress plant diseases and thereby promote good crop plant health, increasing crop yields. Therefore, these thermotolerant Actinomycetota could be used as an alternative to commercial pesticides.

**Table 3:** List of *Streptomyces* spp.—based products as biocontrol agents available in the market worldwide [191].

Commercial Product Name	Organism As Active Substance	Registered As a Microbial Pesticide	Targeted Pest/Pathogen/Disease
Actinovate, Novozymes BioAg Inc., Milwaukee, WI, USA	*S. lydicus* WYEC 108	Canada, USA	Soilborne diseases, viz. *Pythium*, *Fusarium*, *Phytophthora*, *Rhizoctonia*, and *Verticillium*; foliar diseases such as powdery and downy mildew, *Botrytis*, *Alternaria*, *Postia*, *Geotrichum*, and *Sclerotinia*
Mycostop, Verdera Oy, Espoo, Finland	*Streptomyces* K61	EU, Canada, USA	Damping off caused by *Alternaria*, *R. solani*, *Fusarium*, *Phytophthora*, *Pythium* wilt, and root diseases
Mykocide, KIBC Co., Ltd., Yongin, Gyeonggi-do, Republic of Korea	*S. colombiensis*	Republic of Korea	Powdery mildews, grey mold, and brown patch
Bactophil	*Streptomyces albus*	Ukraine	Seed germination diseases

### 5.5. Antitumor Compounds

The novel antitumor drug discovery tends to be accomplished by natural product-producing marine microorganisms. Marine-derived actinomycetes are one of the promising candidates in antitumor compound screening. From 2007 to 2017, *Micromonospora*, *Salinispora*, and *Verrucosispora* were among the top new secondary metabolite producers [192]. In previous publications, the reported compounds were categorised into four groups based on natural product classes: polyketides, alkaloids, peptides, and quinones. Actinomycetes have notable anticancer therapeutic potential, especially those whose products are associated with minimal side effects compared to conventional chemotherapy, such as salinosporamide A [193]. Adriamycin, isolated from *Streptomyces peucetius* [194], inhibits DNA replication and is an anticancer drug. Other effective products for cancer chemotherapeutics are actinomycin D, bleomycin, anthracyclines (daunorubicin), and mitosanes (mitomycin C). These drugs were obtained from *Streptomyces verticillus*, *Streptomyces peucetius*, *S. caespitosus*, and other intrageneric isolates [195]. Marine Actinomycetota compounds with antitumor potential include streptochlorin, actinofuranones, aureoverticillactam, chalocomycin B, cyanosporasides, komodoquinones, nonactin, resitoflavine, sporolides, tetracenomycin D, thiocoraline, t-muurolol, butenolides, echinosporins, rakicidin D, and streptokordin [45,195,196]. Important secondary metabolites from marine actinomycetes with antitumor potential include streptopyrrolidine, cyclo-(l-Pro-l-Met), streptochlorin, lynamicins, marizomib, and thiocoraline [195]. Two examples of novel anticancer metabolites are the compound extracts ULDF4 and ULDF5 derived from *Streptomyces* strains found in Lagos, Nigeria. ULDF4 and ULDF5 show cytotoxicity against human acute myelocytic leukaemia, cervical carcinoma, human gastric carcinoma, breast adenocarcinoma, and human acute promyelocytic leukaemia. ULDF4 and ULDF5 are structurally similar to staurosporine and kigamicin, compounds known to induce apoptosis and necrosis, respectively. Ketomycin is another prospective antitumor compound. Ketomycin suppressed breast carcinoma cell migration and invasion, inhibited nuclear factor kappa-B (NF-κB) activity in upstream signalling by impeding the autophosphorylation of inhibitory-κB kinases alpha (IKK-α) and beta (IKK-β), and minimised the 3D invasion of breast carcinoma cells at nontoxic concentrations [197]. Therefore, ketomycin is an effective antibiotic and a structurally simple antitumor agent for mammalian cells. The search for further antitumor agents also includes the analysis of biosynthetic (BGCs) and chemotherapeutic (CGCs) gene clusters. Complementary anticancer treatments have been discovered in *Streptomyces* via the diverse and variable patterns of the phylogenetic distribution of BGCs and CGCs [195]. These hybrid BGCs and CGCs are prospective sources of novel secondary metabolites and chemotherapeutic agents for pharmaceuticals. The use of *Streptomyces* compounds such as staurosporine, kigamicin, and ketomycin and BGCs/CGCs should be further investigated for developing new antitumor treatments.

Most recent anticancer metabolites were identified from *Streptomyces*. The remaining were found in rare actinomycetes from the genera *Actinoalloteichus*, *Actinokineospora*, *Actinomadura*, *Actinosynnema*, *Amycolatopsis*, *Catenuloplanes*, *Dietzia*, *Microbacterium*, *Micromonospora*, *Nocardiopsis*, *Nocardiopsis*, *Nonomuraea*, *Saccharomonospora*, *Tsukamurella*, *Umezawaea*, *and Verrucosispora* (Table 4).

### 5.6. Antiviral Agents

The outbreaks of the influenza A virus (IAV), severe acute respiratory syndrome coronavirus (SARS-CoV), Middle East respiratory syndrome coronavirus, and, recently, SARS-CoV-2 highlight the need for discovering effective antiviral drugs against respiratory RNA virus. Indeed, the coronavirus disease 2019 caused a global pandemic with high mortality rates worldwide. Natural products derived from microbial sources are still an excellent structural motif for discovering new therapeutics, including antiviral agents [268]. About 50% of the US Food and Drug Administration (FDA)-approved natural product-based drugs are of microbial origin, notably antivirals [269]. Vidarabine (Ara-A) isolated from *Streptomyces antibioticus* was one of the earliest antiviral nucleoside analogues [270]. In addition, several ansamycin antibiotics have shown antiviral properties against diverse infectious [271]. The study of Raveh et al. purified a novel metabolite (an Antimycin A derivative) with significant antiviral activity from *Streptomyces kaviengensis* [272]. This compound showed activity against the Western equine encephalitis virus with a half-maximal inhibitory concentration (IC_50_) of <4 nM. The mode of action revealed the disruption of mitochondrial electron transport and pyrimidine biosynthesis [273]. *Streptomyces citricolor* produced aristeromycin that showed potent anti-IAV activity [274]. Protease inhibitor PISC-2002 from the culture supernatant of *Streptomyces chromofuscus* showed antiviral activity against influenza virus A/Rostock/34 (H7N7) [275]. The microbial-derived FDA-approved anti-parasitic drug ivermectin, a semisynthetic pentacyclic sixteen-membered lactone derived from the soil bacterium *Streptomyces avermitilis*, was recently reported. Ivermectin was shown to be an effective in vitro inhibitor of SARS-CoV-2 replication [276,277].

Most recent antiviral metabolites were identified from *Streptomyces*, with the others identified from *Kutzneria* (Table 5). Our research group discovered the in vitro antiviral activity of spectrotetronates and decatromicins against Dengue virus serotype 2, with a suggested mode of action in preventing viral replication and assembly via inhibiting the viral protease [278].

### 5.7. Other Activities

Our research group also discovered bioactive compounds with other activities: adipocyte differentiation, anti-trypanosomal activity, and androgen receptor (AR) antagonistic activity. Three modified amino acids (jomthonic acids A-C) were isolated from a soil-derived *Streptomyces* sp. BB47, with jomthonic acids A and B inducing adipocyte differentiation [289,290]. Five new anti-trypanosomal macrolides (actinoallolides) were identified from the cultured broth of *Actinoallomurus fulvus* MK10-036. Actinoallolide A showed the best in vitro anti-trypanosomal activity with an IC_50_ of 0.0049 μg mL^–1^ without cytotoxicity [291]. Antarlides A-E were isolated from *Streptomyces* sp. BB47. Antarlide B inhibited the transcriptional activity of wildtype and mutant ARs and could be used as an AR antagonist. In 2020, norditerpenoid k4610422, an inhibitor of testosterone-5α reductase, was identified from *Actinomadura* spp. [292].

## 6. Concepts and Methods to Explore New Bioactive Compounds

Many new antibiotics were identified in massive screening programs during the ‘golden age’ of antibiotic discovery from the late 1940s to the late 1960s. While the supply of new antibiotics has declined over the last decade, the emergence of multidrug-resistant microbial pathogens has increased [293,294]. Therefore, finding new therapeutic active compounds remains of scientific interest.

### 6.1. Exploring New Habitats or Extreme Environments As A Source for Novel Strains

Over the past 50 years, researchers have investigated antibiotics and metabolites from terrestrial microorganisms, but 99% of their metabolites are known compounds [192]. Therefore, the chance of discovering novel secondary metabolites has diminished. Currently, only ~1% of existing actinomycetes can be identified based on DNA analyses. However, actual microbial diversity is much greater than expected [295]. The focus of industrial screening has moved to markers of less exploited genera of rare actinomycetes (e.g., *Actinokineospora*, *Jishengella*, *Micromonospora*, *Nocardiopsis*, *Salinispora*, *Saccharomonospora*, *Saccharopolyspora*, and *Verrucosispora*) [296]. Therefore, the isolation of novel species from diverse ecosystems may result in new unprecedented bioactive compounds produced by them.

Extremophile organisms live in extreme habitats and have unique survival mechanisms to withstand harsh conditions, such as high temperature, extreme pH, salinity, pressure, and aridity [297]. Exploration of unique ecological niches and new isolation methods for novel genera/species of actinomycetes may identify new BGCs. While actinomycetes are commonly present in terrestrial and aquatic ecosystems, studies have focused on isolating them from extreme geographical locations, including oceans [40,93,109,206,298], hot springs [299,300], desert soil [13,14,116,301,302], salt lakes [23,303]. Among these extreme habitats, deserts and the deep sea were the most favourable environments for isolating bioactive compounds with potential applications in medicine from actinomycetes [304]. Marine habitats have received increasing attention in recent decades because the ocean covers > 70% of the earth’s surface and has a unique ecosystem. Marine habitats show extreme light, oxygenation, and temperature conditions. Therefore, microorganisms could adapt to survive in these extreme environments and produce unique secondary metabolites [46]. One of the most important sources of new bioactive compounds is endophytic actinomycetes. The association of actinomycetes with plants constitutes a unique trait conferring specific biological and chemical features. They can potentially produce various valuable metabolites [10,12,18,25,135,191,305].

### 6.2. Genome Mining to Investigate Biosynthetic Potential

Genomic-guided approaches help discover new natural products leading to an increasing number of complete genome sequence projects. The publication of the first complete genome sequence of *Streptomyces coelicolor* showed surprisingly more suspected biosynthetic clusters than expected, indicating that many new bioactive compounds remain to be discovered and exploited [306,307]. Next-generation sequencing has revolutionised the field and dramatically increased the reported genomes for actinomycetes. The complete annotated genome sequences of antibiotic-producing actinomycetes, including *S. avermitilis*, *Saccharopolyspora erythraea*, *S. tropica*, and *Rhodococcus* sp. RHA1 revealed numerous (≥20 potential secondary metabolites) PKS, NRPS, and many other small-molecule biosynthetic pathways [295]. This information indicates that actinomycetes remain promising sources of novel bioactive compounds.

Genome mining primarily aims to identify biosynthetic pathways that produce novel bioactive molecules. Clusters of silent or cryptic biosynthetic genes, usually associated with a known chemical class such as polyketide and NRPS, are scanned in silico to search for consensus motifs and predict possible chemical structures. Developing rapid automatic search algorithms is essential to take full advantage of genome sequence information. Currently, the most widely used software to identify such gene clusters is Antibiotics and Secondary Metabolite Analysis Shell (antiSMASH; https://antismash.secondarymetabolites.org, accessed on 27 July 2023) [308]. Other computational tools, such as CLUSEAN [309], PRISM [310], NP.searcher [311], and ClustScan [312], have been developed to identify secondary metabolite BGCs (smBGCs) within the genome. These bioinformatics tools rely on the highly conserved sequences within the smBGCs to map their location. However, the predicted smBGCs still require extensive laboratory work, such as activating silenced smBGCs and purifying and elucidating potential compounds [313].

**Figure 3:**
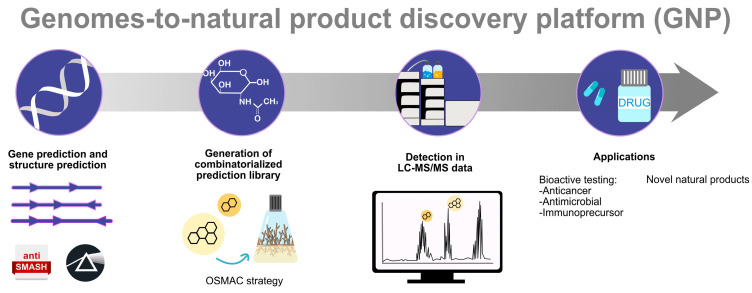
Overview of genome mining approaches. BGCs and their metabolites are predicted from genome sequence data and confirmed by cloning the gene cluster either alone or in a combinational BGC into a heterologous host. Then, liquid chromatography with tandem mass spectrometry (LC-MS/MS) database can be used to predict the compound’s type, and its bioactivity should be confirmed.

The mining of whole genomes from actinomycete species reveals a great genetic potential to synthesise secondary metabolites, including antibiotics. However, only some of these clusters are expressed under standard laboratory conditions. Several methods have been developed to trigger the expression of ‘silent gene clusters’, including optimising fermentation conditions, inducing mutations in ribosomal proteins or RNA polymerase, co-cultivating the antibiotic producer with other microorganisms, and activating or disrupting pathway-specific controlling gene clusters (Figure 3). If a gene cluster of interest cannot be expressed in its original host, it must be cloned into a suitable vector and expressed in a heterologous host. A heterologous expression established an approach for unlocking silent or cryptic gene clusters [314]. Several techniques for assembling and cloning of BGCs have been developed and optimised. Generally, this method includes three steps: (i) cloning of the target BGCs, (ii) engineering of the target BGCs, and (iii) transformation into the selected heterologous host. For cloning large BGCs, a genomic library is constructed by cosmid or fosmid. Transformation-associated recombination is an alternative used for direct cloning of BGCs, where large BGCs can be assembled from multiple overlapping PCR products or restriction fragments isolated from genomic libraries. A silent biosynthetic pathway generating a non-ribosomal peptide from *Saccharomonospora* sp. CNQ-490 was successfully cloned and functionally expressed in *S. coelicolor*. For example, expressing the terpene synthase gene from *S. avermitilis* in an *Escherichia coli* host resulted in the synthesis of a novel tricyclic sesquiterpene: avermitilol [315]. Our research group is working on genome mining of *Actinokineospora bangkokensis* 44EHW^T^ to further investigate bioactive metabolites in addition to thailandins [159].

### 6.3. OSMAC Approach

**Figure 4:**
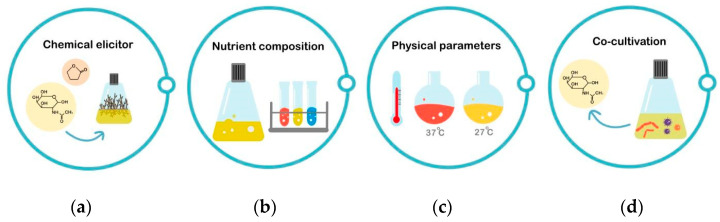
The OSMAC methodology (Inspired by the overview of OSMAC in the review of Romano et al., 2018 [317]). (**a**) Chemical elicitors can trigger silent compounds, (**b**) changing nutrient regimes, (**c**) altering physical parameters such as temperature, and (**d**) Co-cultivation. These methods can improve and trigger the production of many secondary metabolites.

In One Strain-Many Compounds (OSMAC), new approaches unlocking the silent gene clusters are used to improve secondary metabolite production in microorganisms through changes in growth conditions. The name was coined by Zeeck et al. [316]. Its basis is that a single strain of microbes can biosynthesise different compounds when growing under particular conditions. Romano et al. [317] reported that OSMAC strategies are changes in nutrient regimes, physical parameters (e.g., temperature), co-cultivation, and other environmental cues (Figure 4). Variation of the medium is a simple but successful strategy since it directly affects microbial metabolism and differentiation. Changes in nutrient composition, C/N ratio, metal ions, and salt content influence gene expression in the microbial culture. Additionally, a lack of nutrients may induce microbes to attempt to survive by inducing special functional genes. Co-cultivation with other strains may create competitive or favourable conditions. The co-cultivation method possibly alters secondary metabolite production of both known and novel compounds from the silent gene clusters. Mixed cultivation of two strains of fungi (Nos. 1924 and 3893) produced one novel 1-isoquinolone analogue and its methyl ester that was not found with isolated cultivation. Co-cultivation with mycolic acid-containing bacteria was also shown to produce new secondary metabolites in *Streptomyces* [318]. Using γ-butyrolactone (GBL) as a chemical elicitor can induce the biosynthesis of antibiotics in actinomycetes [319]. *N-*acetylglucosamine (GlcNAc) also effectively stimulated bioactive compound production in *Actinokineospora spheciospongiae* sponge-derived actinomycetes [320]. Additionally, the co-cultivation of actinomycetes with fungi led to new compound production: borrelidins J and K and 7-methoxy-2,3-dimethylchromone-4-one [321].

### 6.4. Co-Cultivation Technique

Co-cultivation is the cultivation of two or more microbe strains. In nature, microbes live together in a community. When cultured with other microbes, they may produce a new compound because of nutrient competition or friendly relationships encouraging them to. Additionally, co-cultivation can increase the yield of existing metabolites. For example, the co-culturing of marine *Streptomyces* with human pathogens can induce the production of tree antibiotics and improve activity against Gram-positive human pathogens. Co-culturing of *Streptomyces rochei* MB307, actinomycetes, and the fungi *Rhinocladiella similis* 35 produced new metabolites [321]. A red pigment was produced by *Streptomyces lividans* TK23 during co-cultivation with *Tsukamurella pulmonis* TP-B0596. Moreover, the co-culturing of rare actinomycetes *Umezawaea* sp. RD066910 with mycolic acid-containing bacteria *Tsukamurella pulmonis* TP-B0596 induced the production of umezawamides A and B, new macrolactams toxic to P388 murine leukaemia cells, while umezawamides A can also inhibit *Candida albicans* [185].

### 6.5. Using Chemical Elicitors

Actinomycetes employ renowned extracellular signal molecules such as *N*-acetylglucosamine and γ-butyrolactone to trigger the activation of cryptic secondary metabolism gene operons. The utilisation of chemical elicitors represents a potent approach capable of unlocking the expression of cryptic biosynthetic gene clusters in actinomycetes. Through this method, the production of new or low-yield secondary metabolites is achieved, thereby broadening the diversity, and enhancing the potential for discovering novel bioactive compounds with diverse biological activities [320,322,323]. The review delves into various types of chemical elicitors and their effects on secondary metabolites.

#### 6.5.1. γ-. Butyrolactones and Related Regulators

Organic and low-molecular-weight gamma butyrolactones (GBLs) are chemical agents widely used to trigger secondary metabolite production because bacteria in the *Streptomyces* genus produce them as microbial hormones (autoregulators). These hormones control secondary metabolite production and bacterial morphological changes. GBLs’ major backbone is 2-(1″-oxo or 1 ″hydroxyalkyl)-3-hydroxymethyl butyrolactone. GBLs are classified into three major types based on the characteristic of their side chain: the A-factor type (1″-keto-type), the virginiae butanolide type ((1 ″S)-hydroxy-type), and the IM-2 type ((1 ″R)-hydroxy-type). Choi et al. described that *Streptomyces* and rare actinomycetes possess GBL autoregulators, including *Actinoplanes teichomyceticus* and *Amycolatopsis mediterranei* [324]. Examples of GBL regulation include the production of virginiamycin by *Streptomyces virginiae*, an antibiotic used in the ethanol industry to eliminate bacteria; the production of showdomycin and minimycin by *Streptomyces lavendulae*; and the production of pigmented antibiotics Act and Red by *S. coelicolor*. In previous studies, it was demonstrated that the addition of an exogenous butyrolactone compound led to a significant increase in validamicyn antibiotic production in *Streptomyces hygroscopicus* 5008 [322].

However, in actinomycetes, GBLs are not the sole regulators present. Other types of autoregulators have been reported, such as the pi-factor from *S*. *natalensis* and l-N-methylphenylalanyl-dehydrobutyrine diketopiperazine from *S*. *globisporus* [322]. Goadsporin, a potent 19-amino acid peptide discovered in the culture broth of *Streptomyces* sp. TP-A0584 exhibits remarkably broad elicitation activity on actinomycetota. Goadsporin has the capability to stimulate *S*. *lividans* to produce red pigment and facilitate the process of sporulation [325].

#### 6.5.2. N-Acetylglucosamine (GlcNAc)

GlcNAc is a monosaccharide glucose amide derivative, the secondary amide of glucosamine and acetic acid. It is a component of a biopolymer in the bacterial cell wall, peptidoglycan layer, or murein. GlcNAc is also a chitin monomer, a polymer in the exoskeletons of arthropods, radula in molluscs, and fungi cell walls. Tawfike et al. reported that GlcNAc could activate cryptic gene clusters in sponge-derived rare actinomycete *Actinokineospora spheciospongiae* sp. during cultivation in broth and on solid agar. Four new actinosporins (E–H) were discovered in broth culture. Additionally, fridamycins H and I and actinosporins C, D, and G were produced in solid agar culture [320]. Moreover, their previous study explored the effect of GlcNAc on cultured *Micromonospora* sp. RV43, *Rhodococcus* sp. RV157, and *Actinokineospora* sp. EG49. They found that GlcNAc-induced *Micromonospora* sp. RV43 to produce 3-formylindole and guaymasol, *Rhodococcus* sp. RV157 to produce the siderophore bacillibactin antibiotic surfactin and *Actinokineospora* sp. EG49 to increasingly produce actinosporins E–H [326].

## 7. Future Perspectives on Actinomycetes

Actinomycetes produce diverse bioactive metabolites, many of which play important roles as therapeutic lead compounds. Therefore, their exploration can provide an enormous reservoir of potentially active compounds [63,327,328]. Actinomycetes are distributed across diverse habitats, including terrestrial and marine ecosystems. They can be isolated from soil, water, sediments, plants, and insects [38,329,330,331]. While they already provide various therapeutic drugs available on the commercial market, many members of this bacterial group and, importantly, their potential secondary metabolites with possible immense therapeutic values remain underexplored [328]. New genera and species are generally promising sources of novel bioactive secondary metabolites. Therefore, it is crucial to understand the strains’ biodiversity, to further investigate and identify new genera or species, and to optimise compound production processes [3,4,296]. An investigative approach to screen and select new strains with a potential therapeutic metabolite should be considered and used. In addition, many BGCs are usually found in their genome sequence, and many clusters are silenced. Activating these silent gene clusters may lead to new compound discovery [332,333]. The preliminary identification of the whole genome sequence can help to investigate the biodiversity of strains in various ecosystems and identify their BGCs. Genome-dependent mining can be used to identify the silent BGCs in their analysed genome sequences [334]. Genetic engineering by cloning and expressing the whole BGC in a suitable heterologous host could be an alternative method to activate these silent gene clusters [314,335,336,337]. It is known that one bacterium can produce new metabolites in the presence of other bacteria or fungi. Therefore, co-cultivation approaches have been adopted, and many silent gene clusters have been successfully activated [321,338]. The competition for nutrients and any signal molecules from other microorganisms may elicit new compound production from the target host strain. However, while co-cultivation can activate silent gene clusters, it can sometimes silence others. Therefore, the selection of co-cultivation partners is challenging. A better understanding and elucidation of the key signal in eliciting silent gene cluster expression is necessary to obtain new potential metabolites.

## 8. Conclusions

Despite the huge amount of available research information on exploring actinomycetes and their secondary metabolites, there remains significant potential for discovering new bioactive agents from these microorganisms. Integrative approaches involving new cultivation techniques, chemical elicitor induction, genome mining, and gene cluster activation should be employed to find new compounds with essential functions. Rapid progress in using genetic tools and several approaches for isolating new compounds with novel bioactivity will encourage researchers in this field to obtain promising candidates for fighting against emerging pathogens and various diseases, including non-communicable diseases. Novel genera and novel species of actinomycetes from diverse habitats are still waiting to be discovered. Marine habitats represent an underexplored ecosystem rich in microbial and compound diversity, making their metabolites highly promising for commercial applications. While marine actinomycetes may produce some of the same metabolites as their terrestrial counterparts, they have also demonstrated to be a valuable source of potential novel metabolites, especially in the case of rare marine actinomycetes.

## Figures and Tables

**Table 1:** Examples of antibacterial metabolites identified from Actinomycetota from 2016 to 2023 *.

Organism (s)	Compound Name (s)	Reference (s)
*Actinoallomurus* sp. ID145113, 145206, 145754	Paramagnetoquinones A–C	[66]
*Actinomadura atramentaria* NBRC 14695	Cinnamycin B	[67]
*Actinomadura* sp. KC191	Actinomadurol	[68]
*Amycolatopsis* sp. IRD-009	Pradimicin-IRD	[69]
*Amycolatopsis* sp. M39	Macrotermycins A & C	[70]
*Amycolatopsis* sp. MCC0218	Enceleamycins A–C	[71]
*Amycolatopsis* sp. ML1-hF4	Pargamicins B–D	[72]
*Kibdelosporangium phytohabitans* XY-R10	Maipomycin A (MaiA)	[73]
*Kitasatospora* sp. MG372-hF19	Biospolides C-E	[74]
*Kocuria marina* CMG S2	Kocumarin	[75]
*Micromonospora carbonacea* LS276	Tetrocarcin Q	[76,77]
*Micromonospora harpali* SCSIO GJ089	Icrosporanate A, Tetrocarcin P, Microsporanates B–F	[78]
*Micromonospora chalcea* FIM 02	Rakicidins G–I	[79]
*Micromonospora* sp. UR56 + *Actinokineospora* sp. EG49	Phenazine-1-carboxylic acid, Aestivophoenin C, Methyl saphenate	[80]
*Micromonospora* sp. WMMB-235 + *Rhodococcus* sp. WMMA-185	Keyicin	[81]
*Micromonospora* sp. 5-297	Tetrocarcins N	[82]
*Micromonospora* sp. CA-214671	Phocoenamicin B	[83]
*Micromonospora* sp. RJA4480	Sporalactam B	[84]
*Micromonospora* sp. SCSIO 07395	Microechmycin A	[85]
*Micromonospora* sp. TP-A0468	16-demethylrifamycin S	[86]
*Micromonospora endolithica*	loseolamycins A1, A2	[87]
*Micromonospora* yangpuensis DSM 45577	Yangpumicins F, G	[88]
*Nocardiopsis* sp. LX-1	Nocarpyrroline A, Isoflavonoid E	[89]
*Nocardiopsis* sp. SCA30	1-acetyl-4-4(hydroxyphenyl)piperazine	[90]
*Pseudonocardia carboxydivorans* M227	Branimycins B, C	[91]
*Streptomonospora* sp. PA3	Persiamycin A	[92]
*Streptomyces aculeolatus* PTM-029	Napyradiomycins (3,7,9,10,12)	[93,94]
*Streptomyces aculeolatus* PTM-420	Napyradiomycins (1,2,4–6,8,11)	[93]
*Streptomyces albus* 4N24	Benzanthric acid	[95]
*Streptomyces albus* MAB56	12-methyltetradecanoic acid, Palmitic acid, Tridecanoic acid	[96]
*Streptomyces armeniacus* DSM 43125	Armeniaspirol analogues 1–6, 9–12	[94]
*Streptomyces bacillaris* MBTC38	Lactoquinomycin A	[97]
*Streptomyces californicus* ADR1	Methanoazulen-9-ol, decahydro-2, 2, 4, 8-tetramethyl-stereoisomer (Sesquiterpene)	[98]
*Streptomyces coelicolor* LY001	3-(3,5-dichloro-4-hydroxyphenyl) propanoic acid, 3-(3,5-dichloro-4-hydroxyphenyl) propanoic acid methyl ester, 3-(3-chloro-4-hydroxyphenyl) propanoic acid	[99]
*Streptomyces diacarni* LHW51701	Chlocarbazomycins (CCBs) A–D	[100]
*Streptomyces fradiae* MM456M-mF7	Fradiamines B	[101]
*Streptomyces globisporus* sp. WA5-2-37	Actinomycin X2, Collismycin A	[102]
*Streptomyces globisporus* subsp. globisporus	Globimycin	[103]
*Streptomyces griseoviridis* PU-KB10–4	Mitomycin C	[104]
*Streptomyces hyaluromycini* MB-PO13	Rubromycins CA1, CA2	[105]
*Streptomyces koyangensis* SCSIO 5802	Neoabyssomicins F, G	[106]
*Streptomyces malachitospinus* ITD-35	3-octanone, neopentyl, Isothiocyanate, 2-methyl butyl isothiocyanate	[13]
*Streptomyces microflavus* (MBTI36)	Chromomycin A9, Ap, A2, A3	[107]
*Streptomyces lunaelactis* MM109T	Lunaemycins A, B1, D	[108]
*Streptomyces lusitanus* OUCT16-27	Grincamycin L	[109]
*Streptomyces palmae* CMU-AB204T	Phenyl alkenoic acids C, D Phenyl alkenoic acids E, F	[110]
*Streptomyces qinglanensis* 172205	15*R*-17,18-dehydroxantholipin	[111]
*Streptomyces zhaozhouensis* 208DD-064	Streptopyrroles B, C	[112]
*Streptomyces* sp. 120454	Mayamycin B	[113]
*Streptomyces* sp. 182SMLY	*N*-acetyl-*N*-demethylmayamycin	[114]
*Streptomyces* sp. 7NS3	Emycin A	[115]
*Streptomyces* sp. 8P21H-1	Desulphurzing, Griseoviridin, Griseoviridin	[116]
*Streptomyces* sp. AD-3-6	Nybomycins D	[117]
*Streptomyces* sp. ADI91-18, ADI95-16	Linearmycins	[117]
*Streptomyces* sp. ADI97-07	Clavams	[117]
*Streptomyces* sp. ADI98-12	Griselimycin, Kirromycin	[117]
*Streptomyces* sp. B9173	Flaviogeranin B1, B, Flaviogeranin D, Flaviogeranin C2	[118]
*Streptomyces* sp. CA-271078	Napyradiomycins 2, D1	[119]
*Streptomyces* sp. CPCC 204980	Cervinomycins B1–B4	[120]
*Streptomyces* sp. DSM14386	Svetamycins C, G	[121]
*Streptomyces* sp. FJS31-2	Zunyimycins B, C	[122]
*Streptomyces* sp. GKU 220	Rakicidin F	[123]
*Stretomyces* sp. HCCB11876	Quinomycins I, J	[124]
*Streptomyces* sp. HK-2006-1	Aldgamycin O	[125]
*Streptomyces* sp. HN-A124	Cysrabelomycin	[126]
*Streptomyces* sp. Hu103	Anulamycins A-D	[127]
*Streptomyces* sp. HZP-2216E	23-*O*-butyrylbafilomycin D	[128]
*Streptomyces* sp. IB201691-2A	Baikalomycins A–C	[129]
*Streptomyces* sp. ICN19	Ala-geninthiocin	[130]
*Streptomyces* sp. KCB13F003	Ulleungmycin A, B	[131]
*Streptomyces* sp. KCB14A132	Enamidonins B, C	[132]
*Streptomyces* sp. MM168-141F8	Quadoctomycin	[133]
*Streptomyces* sp. MNP32	Phenol, 3,5-bis(1,1-dimethylethyl)-1,1′-Biphenyl]-2,3′-diol, 3,4′,5,6′-tetrakis(1,1-dimethylethyl)	[134]
*Streptomyces* sp. MUSC 125	Thiophene, 2-butyl-5-ethyl, 1-Heptyn-3-ol, 8-[N-Aziridylethylamino]-2-6,dimethyloctene-2, Pyrrolo[1,2-a]pyrazine-1,4-dion,hexahydro, and 2,4-Dihydroxy-6-propylbenzoic acid	[42]
*Streptomyces* sp. NA06554	Borrelidin J	[135]
*Streptomyces* sp. NA07423	Nagimycins A, B	[136]
*Streptomyces* sp. OPOK_MB_B11	Azalomycin F4a 2-ethylpentyl ester	[137]
*Streptomyces sp.* PBR11	1-Tetradecanol phenol, 2,5-bis(1,1-dimethylethyl n-pentadecanol, 1-nonadecene, and pyrrolo[1,2-a]pyrazine-1,4-dione hexahydro-3-(phenylmethyl).	[138]
*Streptomyces* sp. PNM-9	2-methyl-*N*-(2′-phenylethyl)-butanamide 3-methyl-*N*-(2′-phenylethyl)-butanamide	[139]
*Streptomyces* sp. SBT345	Ageloline A	[140]
*Streptomyces* sp. SCSIO 41399	Isotirandamycin B	[141]
Streptomyces sp. sima1_6	Echinomycin	[137]
*Streptomyces* sp. SN0280	Streptoone A	[142]
*Streptomyces* sp. SM01	Picolinamycin	[143]
*Streptomyces* sp. SS	Sansanmycin Q	[144]
*Streptomyces* sp. Stup16_B49.2	Echinomycin	[137]
*Streptomyces* sp. Stup16_B146	TPU-0037-C	[137]
*Streptomyces* sp. Stup18_J70	Bafilomycin A1 derivative	[137]
*Streptomyces* sp. UICC B-92	4-*O*-glucosyl,1-carboxyl-phenazine	[145]
*Streptomyces* sp. W367A	W367A	[137]
*Streptomyces* sp. XMA39	Strepoxepinmycins A–D	[146]
*Streptomyces* sp. YINM00001	Peperodione, Peperophthalene	[147]
*Streptomyces* sp. ZZ1118	Streptoindoles A–D	[148]
*Streptomyces* sp. ZZ741	Streptoglutarimides A–J	[149]
Streptomycete clade MAR4 CNY-960, CNS-284	Marinocyanins A–F	[150]
*Streptosporangium* sp. SANK 60,501	Muraminomicins A, B, C, D, E1, E2, F	[151]
*Thermoactinomyces vulgaris* ISCAR 2354	Thermoactinoamide A	[152]
*Verrucosispora* sp. FIM06-0036	2-ethylhexyl 1H-imidazole-4-carboxylate	[100]
*Verrucosispora* sp. SCSIO 07399	Kendomycins B–D	[153]

* Most records were published in the PubMed and Web of Science databases from 2016 to 2023.

**Table 2:** Examples of antifungal metabolites identified from Actinomycetota from 2016 to 2023 *.

Organism (s)	Compound Name (s)	Reference (s)
*Actinokineospora bangkokensis* 44EHW^T^	Thailandins A & B	[159]
*Actinomadura* sp. BCC 35,430	Actinomadurone	[161]
*Amycolatopsis* sp. M39	Macrotermycins A & D	[70]
*Acrocarpospora punica* 04107M	Acrocarposporins A, B, D, and E	[162]
*Dermabacter vaginalis* AD1-86	Dermazolium A	[163]
*Micromonospora* sp. UR56 + *Actinokineospora* sp. EG49	Phenazine-1-carboxylic acid, Aestivophoenin c, Methyl saphenate	[80]
*Nocardia abscessus* IFM 10029	Nabscessins A, B	[164]
*Nocardiopsis* sp. LX-1	Isoflavonoid E	[89]
*Pseudonocardia autotrophica* KCTC9441	Polyene B1	[165]
*Saccharothrix yanglingensis* Hhs.015	10-deoxyfungichromin (WH02)	[166]
*Streptomyces albidoflavus* STV1572a	1-heneicosanol	[167]
*Streptomyces albolongus* YIM 101047	19-methoxybafilomycin C1 amide	[168]
*Streptomyces antibioticus* strain 200-09	Kitamycin C	[169]
*Streptomyces caniferus* GUA-06-05-006A	PM100117, PM100118	[170]
*Streptomyces griseoviridis* PU-KB10–4	Mitomycin C	[104]
*Streptomyces hyaluromycini* MB-PO13	Rubromycins CA1, CA2	[105]
*Streptomyces morookaense* AM25	Gloeosporiocide	[171]
*Streptomyces* sp. 1H-XA2	Furamycins I, II	[172]
*Streptomyces* sp. ADI91-18	Linearmycins	[117]
*Streptomyces* sp. ADI95-16	Linearmycins	[117]
*Streptomyces* sp. ADI96-02	Cycloheximide, Galbonolides	[117]
*Streptomyces* sp. ADI96-15	Candicidin	[117]
*Streptomyces* sp. ADI97-07	Galbonolides	[117]
*Streptomyces sp.* ADI98-12	Candicidin	[117]
*Streptomyces* sp. BV410	Staurosporine	[173]
*Streptomyces* sp. CB09030	LOB A, B, H8	[174]
*Streptomyces* sp. CT37	Legonimide 1, 1H-indole-3-carbaldehyde	[175]
*Streptomyces* sp. FX13	Oligomycin A	[176]
*Streptomyces* sp. HAAG3-15	Azalomycin B	[177]
*Streptomyces* sp. KIB-H869	Hygrolidin-type macrolide	[178]
*Streptomyces* sp. MUSC 125	Thiophene, 2-butyl-5-ethyl, 1-Heptyn-3-ol, Pyrrolo[1,2-a] pyrazine-1,4-dion,hexahydro, 9,9-Dimethyl-3, 7-diazabicyclo[3.3.1]nonane, 2,4-Dihydroxy-6-propylbenzoic acid	[42]
*Streptomyces sp.* PBR11	1-tetradecanol, n-pentadecanol, 1-nonadecene, pyrrolo [1,2-a]pyrazine-1,4-dione hexahydro-3-(phenylmethyl)	[138]
*Streptomyces sp.* QHH-9511	6-deoxy-13-hydroxy-8,11-dione Dihydrogranaticin A Granaticin A, B	[179]
*Streptomyces* sp. SN0280	Streptoone B	[142]
*Streptomyces* sp. SY1965	Streptothiazomycin A, Streptodiketopiperazines A, B, [2-hydroxy-1-(hydroxymethyl)ethyl]-2-methoxybenzamide, salicylamide, 4-hydroxymethyl benzoate, Spoxazomicin C	[180]
*Streptomyces* sp. TR1341	Filipin, Fungichromin, Actinomycin X2	[181]
*Streptomyces* sp. TT3	Actinorhodin	[182]
*Streptomyces* sp. YO15-A001	YO-001A	[183]
*Streptomyces albus* CAI-21	Organophosphate	[184]
*Streptomyces coelicolor* LY001	diketopiperazine alkaloids cyclo (l-Phe-trans-4-OH-l-Pro), cyclo(l-Phe-cis-4-OH-d-Pro)	[99]
*Streptomyces palmae* CMU-AB204^T^	Phenyl alkenoic acids A,B, Anguinomycin A, Leptomycin A, Actinopyrone A	[110]
*Streptomyces qinglanensis* 172205	15R-17,18-dehydroxantholipin	[111]
*Streptomycete clade* MAR4 CNY-960 and CNS-284	Marinocyanins A	[150]
*Umezawaea* sp. RD066910 + *Tsukamurella pulmonis* TP-B0596	Umezawamides A	[185]

* Most records were published in the PubMed and Web of Science databases from 2016 to 2023.

**Table 4:** Examples of antitumor metabolites identified from Actinomycetota from 2016 to 2023 *.

Organism (s)	Compound Name (s)	Reference (s)
*Actinoalloteichus hymeniacidonis* 179DD-027	Dokdolipid B	[198]
*Actinomadura* sp. K13-0306	Sagamilactam	[199]
*Actinomadura* sp. K4S16	Nonthmicin, Ecteinamycin	[200]
*Actinosynnema pretiosum* HGF052::*asm18*	Actinosynneptide A, B	[201]
*Amycolatopsis* sp. IRD-009	Pradimicin-IRD	[69]
*Amycolatopsis alba* DSM 44,262	Thioalbamide	[202]
*Catenuloplanes* sp. RD067331 + *Tsukamurella pulmonis* TP-B059	Catenulobactin B	[203]
*Micromonospora* sp. UR56 + *Actinokineospora* sp. EG49	Phenazine-1-carboxylic acid, Aestivophoenin c, Methyl saphenate	[80,204]
*Micromonospora carbonacea* LS276	Tetrocarcin Q	[76]
*Micromonospora zhangzhouensis* HM134^T^	7E,11E)-6-hydroxy-1-isopropyl-11-(methoxycarbonyl)-4-methylene-1,2,3,4,4a,5,6,9,10,12a-decahydrobenzo[10]annulene-7-carboxylic acid	[205]
*Micromonospora matsumotoense* M-412	Paulomycin G	[206]
*Micromonospora aurantiaca* 110B	Isoflavonoid glycosides 1-3	[207]
*Micromonospora chalcea* FIM 02-523	Rakicidins G–I	[208]
*Micromonospora* sp. FIM05328	FW05328-1	[209]
*Micromonospora* sp. HS-HM-036	Naphthalenepropanoic acid analog	[210]
*Micromonospora yangpuensis* DSM 45577	Yangpumicin A	[211]
*Micromonospora yangpuensis* DSM 45577	Yangpumicins F, G	[88]
*Nocardiopsis alba* KM6-1	Isomethoxyneihumicin	[212]
*Nocardiopsis* sp. CG3	Kenalactams A, E	[213]
*Nonomuraea endophytica* GW58/450	Karamomycins B, C	[214]
*Nonomuraea* sp. AKA32	Akazamicin	[215]
*Saccharomonospora* sp. UR22 + *Dietzia* sp. UR66	Saccharomonosporine A	[216]
*Streptomonospora* sp. PA3	Persiamycin A	[92]
*Streptomyces albolongus* YIM 101047	19-methoxybafilomycin C1 amide, 21-deoxybafilomycin A1	[168]
*Streptomyces ardesiacus* 156VN-095	Urdamycins W, X, Grincamycin U	[217]
*Streptomyces bacillaris*	2,4-di-tert-butylphenol	[218]
*Streptomycete clade* MAR4 CNY-960 and CNS-284	Marinocyanins A–F	[150]
*Streptomyces qinglanensis* 172205	15R-17,18-dehydroxantholipin, (3E,5E,7E)-3-methyldeca-3,5,7-triene-2,9-dione, Qinlactone A–B	[111]
*Streptomyces cacaoi* subsp. *asoensis* H2S5	Trienomycins J–L	[219]
*Streptomyces caniferus* GUA-06-05-006A	PM100117, PM100118	[220]
*Streptomyces coelicolor* M1146	Staurosporines M1, M2	[221]
*Streptomyces curacoi* NBRC 12761	Curacozole	[222]
*Streptomyces cyaneofuscatus* M-157	3-Hydroxyquinaldic acid derivative 1	[223]
*Streptomyces griseoviridis* 2464-S5	Prodigiosin R2	[224]
*Streptomyces seoulensis* A01	Ansaseomycins A, B	[225]
*Streptomyces subflavus* subsp. *irumaensis* AM-3603	Bisoxazolomycin A	[226]
*Streptomyces violascens* YIM 100225	Albaflavenoid	[227]
*Streptomyces violascens* YIM100212	Fusicomycin A, B, Isofusicomycin A	[228]
*Streptomyces* sp. 166	Sekgranaticin	[229]
*Streptomyces* sp. 182SMLY	*N*-acetyl-*N*-demethylmayamycin, Streptoanthraquinone A	[230]
*Streptomyces* sp. 1H-GS5	Spectinabilin derivative 1	[231]
*Streptomyces* sp. A65	Streptocarbazoles C, 2040-epi-K252d, 0-epi-K252d	[232]
*Streptomyces* sp. AD-3	Nybomycins D	[233]
*Streptomyces* sp. ADI92-24	Tomaymycin	[117]
*Streptomyces* sp. ADI95-17	Naringenin	[117]
*Streptomyces* sp. ADI96-02	Echinomycin	[117]
*Streptomyces* sp. ADI96-15	Antimycin, Lobophorins	[117]
*Streptomyces* sp. ADI97-07	Neocarzilin, Antimycin	[117]
*Streptomyces* sp. ADI98-10	Actinomycin	[117]
*Streptomyces* sp. B9173	Flaviogeranin B1, B, C2, D	[118]
*Streptomyces* sp. BB47	Antarlides F–H	[234]
*Streptomyces* sp. CHQ-64 Δ*rdmF*	Geranylpyrrol A, Piericidin F	[235]
*Streptomyces* sp. CMAA 1527	Cinerubin B	[15]
*Streptomyces* sp. CMAA 1653	Actinomycin V	[15,120]
*Streptomyces* sp. CPCC 204,980	Cervinomycins B1-B4	[120]
*Streptomyces* sp. DT-A61	9-hydroxy-K252c, 3-hydroxy-K252c, 3-hydroxy-7-methoxy-K252c, Nacetylholyrine A, 3-hydroxyholyrine A, 3′-*O*-demethyl-4′-*N*-demethyl-4′-*N*-acetyl-4′-epi-staurosporine, Streptocarbazoles D, E	[236]
*Streptomyces* sp. EGY1	Sharkquinone	[237]
*Streptomyces* sp. GIC10-1	Bafilomycin M	[238]
*Streptomyces* sp. GKU 220	Rakicidin F	[123]
*Streptomyces* sp. HF-11225	Nivelactam B	[239]
*Streptomyces* sp. HN-A124	Cysrabelomycin	[126]
*Streptomyces* sp. HNA39	Cyclizidines B–I	[240]
*Streptomyces* sp. HS-NF-1178	218-seco-lankacidinols A, B	[241]
*Streptomyces* sp. HS-NF-780	9-methylstreptimidone 2-α-d-glucopyranoside, ydroxyiso-9-methylstreptimidone	[242]
*Streptomyces* sp. HZP-2216E	23-*O*-butyrylbafilomycin D	[81,128]
*Streptomyces* sp. ICN19	Ala-geninthiocin	[130]
*Streptomyces* sp. IFM 11490	Elmenols G	[243]
*Streptomyces* sp. KCB13F003	Ulleungdin	[244]
*Streptomyces* sp. KCB13F030	Ulleungoside	[245]
*Streptomyces* sp. KIB-H1318	Phenoxazinone-related alkaloid 2	[246]
*Streptomyces* sp. KIB-H714	Actinomycin Z6	[247]
*Streptomyces* sp. M268	Kiamycin B	[248]
*Streptomyces* sp. MSB090213SC12	Neothioviridamide	[249]
*Streptomyces* sp. MUSC 125	8-[N-Aziridylethylamino]-2-6,dimethyloctene-2	[42]
*Streptomyces* sp. N1510.2	Strepantibin D	[250]
*Streptomyces* sp. N1510.2	Strepantibins A–C	[251]
*Streptomyces* sp. NA4286	Murayaquinone D	[252]
*Streptomyces* sp. NEAU-L3	Tetracenoquinocin A	[253]
*Streptomyces* sp. OPMA00071	JBIR-150	[254]
*Streptomyces* sp. PU-14G	Puromycins C, D	[255]
*Streptomyces sp.* PU-KB10-4	4-hydroxycinnamide	[104]
*Streptomyces* sp. Q22	Bagremycins C	[256]
*Streptomyces* sp. RAI364	Curromycin A	[257]
*Streptomyces* sp. RK88	Opantimycin A	[258]
*Streptomyces* sp. SBT345	Strepoxazine A	[259]
Streptomyces sp. SCSIO 03032	Indimicins F, G, Spiroindimicins G, H	[260]
*Streptomyces* sp. SCSIO 1666/17C4 (engineered)	l-rhodinose-l-rhodinose-2-deoxy-l-fucose-10-decarbomethoxy-ε-rhodomycinone	[219]
*Streptomyces sp.* SCSIO 40063	Piericidins A5, G1	[261]
*Streptomyces* sp. SCSIO 41399	Aranciamycin K, Isotirandamycin B	[141]
*Streptomyces* sp. SS13I	Ephyromycins B, C	[262]
*Streptomyces* sp. SS17F	Thioquinomycins A, C, D	[263]
*Streptomyces* sp. SSA28	Cytosaminomycin E	[264]
*Streptomyces* sp. TR1341	Filipin, Fungichromin, Actinomycin X2	[181]
*Streptomyces* sp. XMA39	Strepoxepinmycins A–D	[146]
*Streptomyces sp.* W2061	Rubiflavin G Photorubiflavin G, E	[265]
*Streptomyces sp.* YIM S01863	Jiangchuanmycin	[266]
*Streptomyces* sp. ZZ741	Streptoglutarimides H	[149]
*Streptomyces* OPOK_MB_A9	Milbemycin-α8	[137]
*Streptomyces* sp. shell-016	Shellmycin A–D	[267]
*Umezawaea* sp. RD066910 + *Tsukamurella pulmonis* TP-B0596	Umezawamides A & B	[185]
*Verrucosispora* sp. SCSIO 07399	Kendomycins B-D	[153]

* Most records were published in the PubMed and Web of Science databases from 2016 to 2023.

**Table 5:** Examples of antiviral metabolites identified from Actinomycetota from 2016 to 2023 *.

Organism (s)	Compound Name (s)	Reference (s)
*Actinomadura* sp. 2EPS	Decatromicins	[278]
*Kibdelosporangium persicum*	Persicamidines A–E	[279]
*Kutzneria albida* DSM 43870	Huimycin	[106,280]
*Streptomyces kebangsaanensis* WS-68302	Napyradiomycin A4, A80915 H	[281]
*Streptomyces jiujiangensis* NBERC-24992	Virantmycins D–G	[282]
*Streptomyces bacillaris*	Zelkovamycins F, G	[283]
*Streptomyces koyangensis* SCSIO 5802	Neoabyssomicins F, G	[106,284]
*Streptomyces* sp. AM-2504	Virantmycins B	[284,285]
*Streptomyces* sp. CPCC 200267	Geninthiocins E, F	[286]
*Streptomyces* sp. HK18	Xiamycins D	[285,287]
*Streptomyces* sp. JA74	Dihydromaniwamycin E	[288]
*Streptomyces* sp. SMU 03	dichloromethane extracts (DCME)	[287]

* Most records were published in the PubMed and Web of Science databases from 2016 to 2023.
